# RETRACTION: Circ_0081054 Facilitates Melanoma Development via Sponging MiR‐637 and Regulating RAB9A

**DOI:** 10.1111/srt.70204

**Published:** 2025-06-22

**RETRACTION**: X. Li, Y. Kong, H. Li, M. Xu, M. Jiang, W. Sun, and S. Xu, “Circ_0081054 Facilitates Melanoma Development via Sponging MiR‐637 and Regulating RAB9A,” *Skin Research and Technology* 29, no. 5 (2023): e13313, https://doi.org/10.1111/srt.13313.

The above article, published online on 3 May 2023 in Wiley Online Library (wileyonlinelibrary.com), has been retracted by agreement between *Skin Research and Technology;* and John Wiley & Sons Ltd. The article has been accepted for publication following an inadequate peer review process. Subsequent to publication it was observed that the rationale for the selection of Circ_0081054 for further investigation in melanoma development is not explained in the article and lacks support from existing literature. Furthermore, the interaction between miR‐637 and RAB9A appears not reproducible with the stated available tools. Accordingly, the article is retracted as the editors consider its conclusions to be invalid. The authors have been informed of the decision of retraction but were unavailable for final confirmation.

